# In Vitro Degradation of Collagen-Based Membranes for Guided Bone Regeneration After Zn-Ions or Doxycycline Functionalization

**DOI:** 10.3390/polym16223109

**Published:** 2024-11-05

**Authors:** Cristina Vallecillo, María T. Osorio, Nuria Infante, María Jesús Ávalos, Marta Vallecillo-Rivas, Christopher D. Lynch, Manuel Toledano

**Affiliations:** 1 Faculty of Dentistry, Colegio Máximo de Cartuja s/n, University of Granada, 18071 Granada, Spain; cvallecillo@ugr.es (C.V.); mtoleosorio@gmail.com (M.T.O.); e.ninfanten01@go.ugr.es (N.I.); mjavalosmarin@gmail.com (M.J.Á.); toledano@ugr.es (M.T.); 2 Restorative Dentistry, University Dental School & Hospital, University College Cork, Wilton, T12 E8YV Cork, Ireland; chris.lynch@ucc.ie; 3 Biosanitary Research Institute, 18012 Granada, Spain

**Keywords:** collagen-based membranes, porcine, dermis, pericardium, zinc, doxycycline, guided tissue regeneration

## Abstract

Collagen-based membrane is the most commonly used biomaterial for guided bone and tissue regeneration; however, its barrier function can be threatened by its rapid degradation pattern, affecting the success of the regeneration process. Differences in the origin and functionalization of the membrane to obtain better properties can alter the degradation rate. The objective of this study was to examine the biodegradation pattern of two commercially available collagen membranes (Jason^®^ and Collprotect^®^) manufactured using porcine pericardium or dermis, doped or not with zinc-ions or doxycycline, in a period up to 21 days. The membrane specimens were subjected to hydrolytic and bacterial degradation tests. The different immersion times were carried out from 12 h up to 21 days. At each time point, quantitative measurements of thickness and weight were made using a digital caliper and an analytic microbalance, respectively. ANOVA and Student–Newman–Keuls tests were carried out for comparison purposes (*p* < 0.05). The differences between time-points within the same membranes and solutions were assessed by pairwise comparisons (*p* < 0.001). Unfunctionalized Jason membrane made of porcine pericardium attained the highest resistance to both degradation tests. The functionalization of the membranes did not alter the biodegradation patterns. All the membranes completely degraded before 48 h in the bacterial collagenase solution, which was the most aggressive test.

## 1. Introduction

Collagen is considered one of the most useful biomaterials in the field of oral and maxillofacial surgery. Its biological characteristics, such as biodegradability and weak antigenicity, make collagen an excellent biocompatibility and safety resource for medical applications [1,2,3]. For these purposes, collagen, presented as type I and III, is mainly obtained from bovine or porcine origin. The dermis, pericardium, and Achilles tendons are the most common extraction areas [4]. Collagen-based biomaterials constitute a set of essential materials in guided tissue regeneration (GTR) and guided bone regeneration (GBR) [2,5]. When it comes to GBR/GTR techniques, collagen is given the form of a membrane. The use of a membrane during defect healing is based on the principle of compartmentalization described by Dahlin in 1988 [6]. Achieving a barrier that keeps the blood clot or graft in the defect allows cells responsible for forming bone to rebuild the lost tissue and prevents the invasion of unwanted cells, such as epithelial tissue cells [6,7].

Membranes for GBR and GTR techniques must satisfy the requirement of possessing an acceptable degradation profile to inversely match the rate of new tissue formation [7,8]. An ideal biomaterial barrier should remain practically intact and in place for an extended period until bone regeneration occurs and subsequently integrates with the surrounding soft tissue. Membranes have been accepted to take 4 weeks to reach structural integrity in periodontal regeneration, whereas in bone tissue regeneration up to 6 months is recommended [2,9,10]. For this reason, the volumetric stability of a membrane over time is a fundamental aspect to consider in GBR and GTR procedures [11,12]. Nevertheless, due to the fairly short barrier functionality of native collagen, these membranes have certain limitations. Collagen-based membranes present an unforeseeable reabsorption time and kinetic of degradation [12,13]. Collagen membranes are rapidly degraded by tissue-specific proteases, collagenases, and enzymatic activity mediated by macrophages and polymorphonuclear leukocytes [4,14,15]. It represents a key drawback in regenerative procedures when a collagen-based membrane, due to its rapid degradation kinetics, does not remain long enough, jeopardizing the success of the intervention [7,8,16]. Demands on membrane stability over time increase even more when large volume defects are regenerated, where a longer healing time is needed [4].

Researchers have focused on how to slow down membrane degradation and lengthen its barrier function for longer. Making improvements in the structure of the membrane and thickness, which depend on the origin of collagen, the obtaining method, and the manufacturing processes, can overcome such drawbacks [16]. The strength of collagen fibrils to decomposition is directly connected to the density of intermolecular cross-links, since it can make it difficult for the hydrolytic water molecule to access [4,17]. When the collagen membrane is cross-linked, its degradation rate slows down; however, such processes have been shown to alter the response of bone-forming cells and tissue integration [7,16]. Other characteristics such as porosity, thickness, and weight have also been mentioned as being responsible for altering degradation patterns [12,18]. Collagen tissue structure varies greatly among marketed membranes, leading to different kinetics of degradation, which impact clinical outcomes [19,20].

Nowadays, there is an increasing interest in developing membranes that, in addition to their occlusive and barrier function, actively participate in the healing and regeneration process [21,22]. Collagen membranes have shown the potential to further improve regenerative properties as effective carriers of active factors [23]. Membranes are functionalized with bioactive components or agents that interact with cells or release drugs [24]. Therefore, as long as the membrane remains in the regeneration area, far from being just a passive barrier, it creates a different chemical context, thus improving its osteoconductive properties [24]. Among the agents used to dope the membranes are zinc-ions and doxycycline. Zinc is a microelement that plays a key function in the immune and nervous system, in addition to intervening in physiological and metabolic processes [25]. Zinc influences the regeneration of both hard and soft tissues in addition to potentially contributing to slower collagen degradation [26]. Certain concentrations of zinc-ions slow and regulate the degradation of the collagen matrix due to the influence on the bioactivity of matrix metalloproteinase (MMP) and the formation of multinucleated giant cells (MNGCs) [27]. It has also been shown that zinc has antimicrobial properties since it can inhibit bacterial grouping in biofilm [28]. Doxycycline is a second-generation semisynthetic tetracycline that appears to improve the maturation and osteogenic capacity of cells, in addition to being an antibacterial agent [29,30]. Doxycycline is an antimicrobial drug that is effective in the treatment of oral infections [31]. This drug has shown antimicrobial, anti-proteolytic, and anti-collagenolytic properties through its MMP-inhibiting activity [32]. Incorporating these antibacterial medicaments with the functionalization method has become a strategy used by numerous researchers to partially surpass the disadvantage of fast degradation caused by bacterial infection [33,34]. It has been proven that doping non-collagen membranes with both zinc-ions and doxycycline increases membranes’ bioactivity without apparently causing changes in their mechanical properties [35]. Notwithstanding, it is unclear whether doping collagen-based membranes with agents can positively or negatively influence the resistance to degradation.

Various types of collagen membranes with different biodegradability have been marketed to meet the needs of tissue and bone regeneration [2]. The resorption pattern of commercially accessible collagen-based barriers can vary widely even more if they are modified. Currently, there are no studies available in the literature on the degradation of doped collagen membranes. Carrying out in vitro studies can provide insights into the influence of the characteristics and composition of the membrane on the duration of the degradation process. Through dimensional and mechanical measurements, it is possible to compare different collagen membranes subjected to the same test conditions. Studying the behavior of these membranes, even when doped, allows us to increase our knowledge of these products. It also helps clinicians in selecting the most suitable membrane considering the specific clinical scenario and provides a useful reference for optimizing manufacturing towards specific performance. The aim of this study was to investigate the biodegradation pattern of two commercially available collagen membranes, doped or not with zinc-ions and doxycycline, in a period of 1 to 21 d. The null hypotheses that were examined were that: (i) The membranes do not have the same degradation kinetics over time; and (ii) the membranes do not respond to the hydrolytic and bacterial collagenase degradation test in a similar way.

## 2. Materials and Methods

### 2.1. Description of the Membranes

Two experimental GBR and GTR collagen-based membranes were tested. These heterologous membranes are CE-certified for clinical use in dentistry and are therefore commercially accessible. The resorbable membranes investigated were: (1) Jason^®^ (botiss biomaterials GmbH, Zossen, Germany), a thin porcine pericardium collagen membrane designed and fabricated for bone tissue regeneration—Jason^®^ membrane acts as a natural long barrier due to the composition and shape of collagen fibers of pericardial origin; (2) Collprotect^®^ (botiss biomaterials GmbH, Zossen, Germany), a porcine dermis cross-linked membrane based on type I and III collagen and recommended for both soft tissue and bone regeneration. Furthermore, the collagen from Collprotect^®^ membrane contributes to the early stabilization of the wound, promoting natural healing. The characteristics of both membranes are available in Table 1.

### 2.2. Membranes Functionalizacion

Eighteen squared pieces (10 × 10 mm^2^) of each membrane type, Jason^®^ and Collprotect^®^, were prepared. Six samples of each membrane brand were functionalized with doxycycline and six others with zinc-ions. For that purpose, 2 mg/mL of sterile phosphate buffered saline (PBS) (pH~7) (Sigma, St. Louis, MO, USA) solutions of doxycycline hyclate (Sigma, St. Louis, MO, USA) and zinc chloride (Sigma, St. Louis, MO, USA) were prepared. An amount of 50 µL of each of these solutions was added to the selected membrane samples, then they were preserved at 37 °C until complete solvent evaporation and placed in a vacuum chamber for complete dehydration. Hence, six groups of membranes were obtained with six samples each: (a) Unfunctionalized Jason^®^ membrane (JM); (b) Jason^®^ functionalized with doxycycline (Dox-JM), (c) Jason^®^ functionalized with zinc-ions (Zn-JM), (d) Unfunctionalized Collprotect^®^ Membrane (CM); (e) Collprotect^®^ functionalized with zinc-ions (Zn-CM); and (f) Collprotect^®^ functionalized with doxycycline (Dox-CM). The mean thickness and standard deviation (SD) in millimeters of the Jason^®^ and Collprotect^®^ membrane specimens before doping were 0.021 (SD 0.03) and 0.099 (SD 0.07), respectively, and after functionalization the mean thickness was 0.019 (SD 0.02) for Jason^®^ and 0.069 (SD 0.07) for Collprotect^®^.

### 2.3. Degradation Assay

Three samples of each experimental group were randomly assigned to two different degradation solutions (PBS or *Clostridium histolyticum* bacterial collagenase) and then immersed separately in 2 mL of each solution (n = 3) [15,36].

Hydrolytic degradation test: membranes were exposed to a phosphate buffer saline solution (PBS) at a temperature of 37 °C [37].

Bacterial collagenase resistance test: membranes samples were immersed in a collagenase solution from *C. histolyticum* bacteria type V (Sigma-Aldrich, St Louis, MO, USA). This solution induces a breakdown of tissues due to the aggressive activity of different enzymes like collagenase, clostripain, aminopeptidase, and both neutral and non-specific proteases. The biological activity of collagen is ≥125 CDU/mg solid. The collagenase solution was prepared by dissolving 2 UI/mL of collagenase in 50 mM Tris HCl (pH of 7.4) with 10 mM CaCl2 [10,38].

The degradation solutions were suctioned and renewed every 48 h [39]. To perform the degradation tests, the membranes were exposed to both degradation tests during the different immersion periods: 12 h, 24 h, 48 h, 7 d, and 21 d. After each immersion period, the membranes were removed from the medium and placed in a vacuum chamber for complete dehydration. Once the samples were dried, the degradation was evaluated. An analytic balance (A&D-Instruments, Frankfurt, Germany) was employed to weight (W) measurements. Using an antivibratory table, the accuracy of this device was 0.0001 g. Additionally, random sample positions were selected to analyze the membrane thickness (Th). The measurements were performed with a digital caliper (Mitutoyo 293-561, Tokyo, Japan) [15,36].

### 2.4. Statistical Analysis

The Kolmogórov–Smirnov test was employed to establish the normal distribution of data (*p* > 0.05). Hence, parametric tests were employed. Thickness and weight were classified as dependent variables. The type of membrane, degradation solution, and immersion time were considered independent variables. The influence of the independent variables on the dependent variables was assessed by multiple ANOVA models. Likewise, analyses of interaction were performed. To calculate the differences between the degradation solutions and materials, post-hoc comparisons of ANOVA and Student–Newman–Keuls were carried out. To perform these comparations, thickness and weigh measurements were converted into percentage of variation using the equation described as follows:

Percentage of loss = [(X0 − Xt)/X0] × 100, (1)

The initial thickness or weight measurement was considered as X0, while Xt was the sample’s thickness or weight at a respective time point (t).

Pairwise comparisons were also conducted using Bonferroni’s correction to detect variances between immersion time-points within the same membrane type and solution experimental group. The sgnificance was set at *p* < 0.05 for all the analyses except for pairwise comparisons, where the significance was considered at *p* < 0.001. A statistical analysis was performed using the SPSS 25.0 (SPSS Inc., Chicago, IL, USA) software package.

## 3. Results

**Figure 1:**
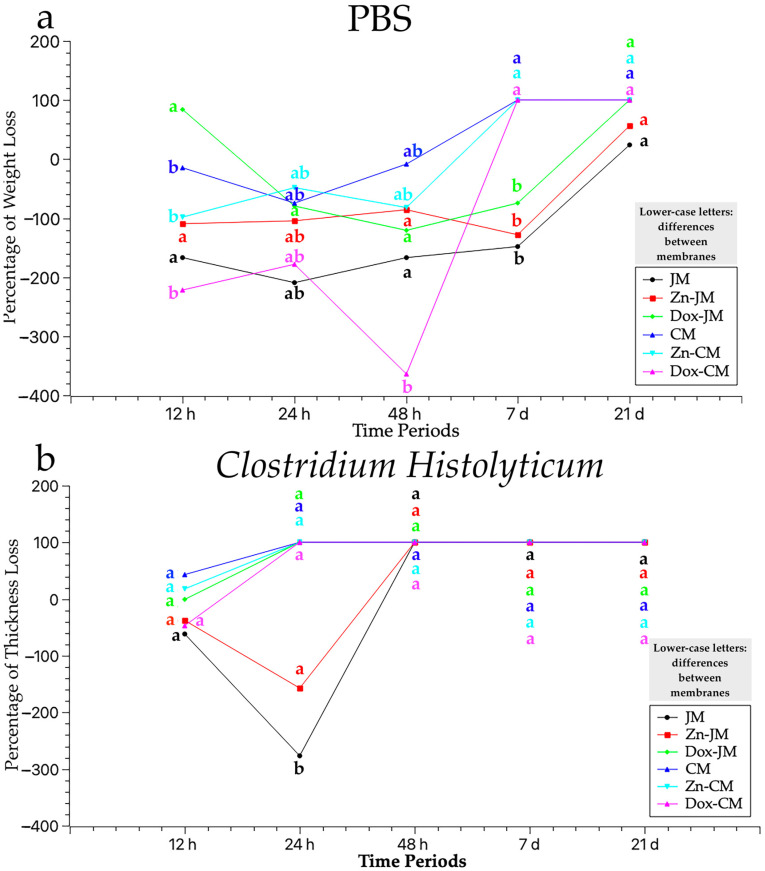
Mean percentage values of thickness loss of the studied matrices, at the different time periods (baseline to 21 days) for the two degradation tests: (**a**) PBS and (**b**) *C. histolyticum* collagenase. Lower-case letters indicate statistical significance for differences between membranes when using the same degradation test. Multiple comparisons were carried out using Student–Newman–Keuls tests. Significance was set at *p* < 0.05. Statistically significant differences between degradation tests were always found when considering membrane and storage time.

The thickness measurements, expressed in millimeters (mm), of the six samples tested (JM, Zn-JM, Dox-JM, CM, Zn-CM and Dox-CM), immersed in two solutions (PBS and *C. histolyticum* collagenase) during different time periods (12 h, 24 h, 48 h, 7 d and 21 d), are represented in Table 2. The percentages of thickness loss across the different time point and degradation tests are illustrated in Figure 1. Statistically significant differences between the degradation tests were always found when considering membrane and storage time (Figure 1).

### 3.1. Thickness Assessment After PBS Degradation Assay

Figure 1a represents the different degradation responses, in terms of the thickness loss (expressed in percentage) of the tested matrices after being subjected to the PBS degradation test. After 12 h of storage, all the membranes increased their thickness except for Dox-JM, which was the only one that suffered a thickness loss, close to 90%. At the 24 h time point, all the membranes gained thickness compared to time zero. Moreover, all the studied samples did not present a loss of thickness with respect to the 12 h time point except Dox-CM and Zn-CM. Both membranes suffered a thickness loss close to 50% in comparison to the 12 h time-point study. At 48 h, the thickness loss trend was: Dox-CM < JM < Dox-JM < Zn-JM < Zn-CM < CM. The three Collprotect^®^ membranes did not exceed 7 days of storage and were entirely degraded. Finally, after 21 days of storage, the Jason membranes resisted the degradation test. Dox-JM had the highest loss of thickness and JM the lowest, while Zn-JM reached an intermediate performance between them.

In general terms, none of the membranes studied experienced thickness losses but rather gained up to 7 days of storage. CM, Zn-CM and Dox-CM were completely degraded before completing the 7 days of immersion and JM, Zn-JM and Dox-JM resisted all the study times.

### 3.2. Thickness Assessment After C. histolyticum Collagenase Degradation Assay

After 1 h of immersion, CM showed a higher loss of thickness than the others, almost 50%, and Zn-CM also suffered an important loss in thickness. Zn-CM maintained its thickness while the rest of the membranes increased in thickness. Only two membranes, Zn-JM and JM, resisted 24 h of storage; the rest of the membranes completely degraded.

Overall, the obtained results suggest this degradation assay is the most aggressive to the studied membranes (Table 2). None of the matrices lasted 48 h of immersion.

**Figure 2:**
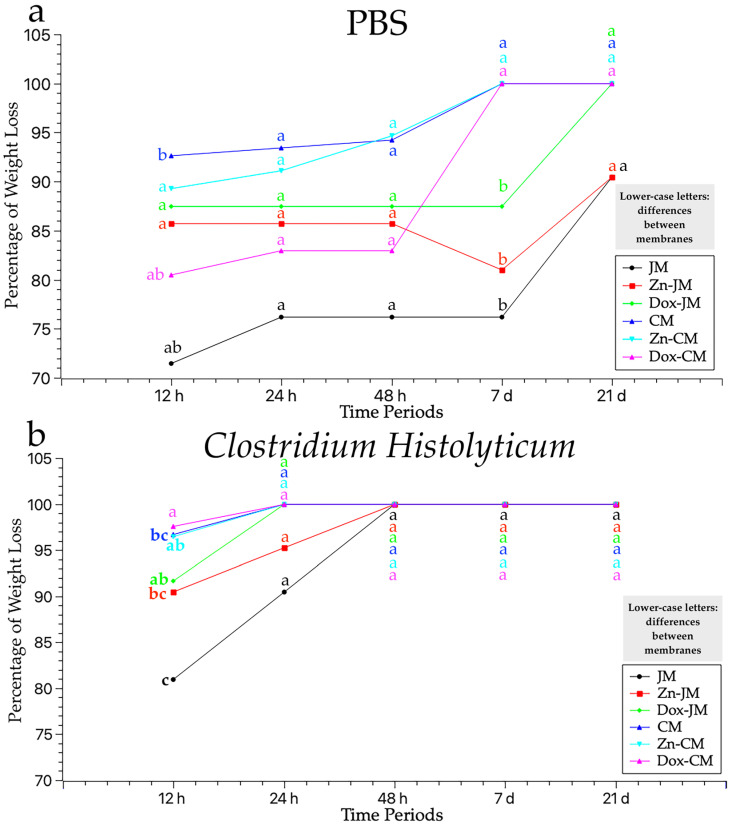
Mean percentage values of weight loss of the studied matrices, at different times periods (baseline to 21 days) in the two degradation tests: (**a**) PBS and (**b**) *C. histolyticum* collagenase. Lower-case letters indicate statistical significance for differences between membranes when using the same degradation test. Multiple comparisons were carried out using Student–Newman–Keuls tests. Significance was set at *p* < 0.05. Statistically significant differences between degradation tests were always found when considering membrane and storage time.

The weight measurements, expressed in grams (g), of the six samples tested (JM, Zn-JM, Dox-JM, CM, Zn-CM and Dox-CM), immersed in two solutions (PBS and *C. histolyticum* collagenase) during different time periods (12 h, 24 h, 48 h, 7 d and 21 d), are represented in Table 3. The weight loss percentages across different measurement times and degradation tests are illustrated in Figure 2. Statistically significant differences between the degradation tests were always found when considering the membrane and storage time (Figure 2).

### 3.3. Weight Assessment After PBS Degradation Assay

After 12 h of immersion, all the membranes experienced a marked weight loss, as shown in Figure 2a. All the membranes, except for JM, suffered a percentage of weight loss greater than 75%. The trend was as follows: JM > Dox-CM > Zn-JM > Dox-JM > Zn-CM > CM. This trend was maintained after 24 h of storage and after 48 h. At the 7 day time point, CM, Zn-Cm and Dox-CM completely degraded while JM, Zn-JM and Dox-JM resisted the degradation test with weight loss percentages similar to those of previous study times. After 21 days, JM and Zn-JM surpassed immersion in PBS medium while Dox-JM did not reach the 21-day study period.

Overall, all the membranes tested showed important weight changes across the different time periods (Table 3). Only two membranes, JM and Zn-JM, completed all the immersion time periods in PBS medium.

### 3.4. Weight Evaluation After C. histolyticum Collagenase Degradation Assay

All the samples suffered a weight loss close to 75% before completing the 12 h of immersion. The tendency was as follows: Dox-CM > CM > Zn-CM > Dox-JM > Zn-JM > JM. After 14 d of immersion, just Zn-JM and JM resisted the degradation test. Zn-JM, CM, Zn-CM and Dox-CM completely degraded. No membrane reached 48 h of immersion.

In general terms, the lowest weight values were obtained in all the time periods after being subjected to this solution, suggesting it is the most aggressive one (Table 3). All the membranes suffered a strong weight loss after the first immersion for 12 h and none of them completed the 48 h of immersion.

## 4. Discussion

This paper sets out the results obtained after confronting two commercially available membranes, doped or undoped with zinc-ions and doxycycline, immersed in two different media, PBS and *C. histolyticum*, for distinct time periods. Once the results had been obtained, it could be stated that the membranes do not behave equally in terms of degradation. Unfunctionalized Jason was the membrane that best resisted the degradation tests, as shown by the thickness and weight values (Figure 1 and Figure 2).

Depending on the origin and structure of collagen, the properties of collagen membranes may vary [40]. Overall, the study results have demonstrated a better response to degradation by functionalized and non-functionalized Jason^®^ membranes than Collprotect^®^ membranes. In this study, the specimens were subjected to in vitro degradation tests. In vitro conditions make it possible to evaluate the membranes’ reaction without the interaction or influence of the inflammatory response from the host tissue. The behavior of the membranes in different media will depend on the membranes’ composition and characteristics. Even when both membranes are obtained from porcine collagen, the site from which the collagen is extracted could be responsible for the different behaviors when facing degradation testing. The pericardial membranes (JM, Zn-JM and Dox-JM) used in this study are composed of collagen fibers with a multidirectional orientation, creating multiple links in a comb-shaped structure [2,7]. The pericardium, thanks to its dense collagen structure, presents rigidity and resistance to appease the forces of the cardiac muscle [41]. Differently, the homogenous dermal-derived collagen membranes (CM, Zn-CM and Dox-CM) present an open porous three-dimensional structure. The open pores provide a less dense matrix that facilitates the migration of blood vessels. Previous research has shown that a higher collagen density, with thicker fibers and a more compact structure result in slower degradation [42,43]. This could explain the greater resistance to degradation of pericardial membranes, with more dense and interconnected fibers, compared to dermal membranes with a less compact three-dimensional structure. Shi et al. [40] found that the degradation rate is related to the collagen’s source and composition. The aforementioned authors, after their research, recommended porcine dermal membranes as the preferred clinical indication with a shorter degradation time; however, no porcine pericardium membrane was considered in this study [40]. According to our results, Vallecillo-Rivas et al. [12] compared the influence of the collagen source on degradation patterns. They found that the porcine pericardium membrane was the most resistant to the different degradation tests [12]. Rothamel et al. [44] faced a porcine pericardium membrane against a porcine dermal origin, finding a slightly faster degradation (4 to 8 weeks) of the dermal membrane in comparison to the pericardium membrane (8 to 12 weeks). The authors also reported comparable results between both membranes in terms of cellular integration, bone formation and immune response [44]. Interestingly, and in contrast to these results, Bornert et al. [8] stated similar degradation rates between Jason^®^ and Collprotect^®^; however, Jason^®^ membrane achieved a higher biocompatibility score with a higher frequency of lymphocytes at implantation sites [8]. At this point, the authors speculated as to whether this faster degradation of the Jason^®^ membrane could be associated with a greater immune response [8]. Ramos et al. [45], who also compared porcine dermis membranes with porcine pericardial membranes, found a more orderly and physiological repair process for the dermal membrane, while the porcine pericardium membrane showed an accelerated and not constant repair process. Another aspect to consider, in addition to the origin and structure, is the initial thickness.

Several studies have investigated the impact of the initial thickness of the collagen membrane on degradation [4,36]. After investigations it has been observed that collagen degrades at the same rate; nonetheless, possessing a double thickness or denser collagen barrier can extend the resorption time [4,36]. In this study, the initial thickness of the membranes was not related to a longer resorption time. It should be stressed that the initial thickness of Jason^®^ membrane is non-uniform, as reported by the manufactures (from 0.05 mm to 0.35 mm). The pericardial membranes degraded more slowly when compared to those of dermal origin. The greater initial thickness of Collprotect^®^ membrane is the result of the arrangement of collagen in a three-dimensional disposition rather than a higher collagen content. Variations in the origin and composition of the membrane lead to differences in the membrane’s degradation rate over time [19]. Thus, the first null hypothesis is rejected.

Notionally, the collagen-based membrane’s degradation speed should coincide with the rate of bone formation, with the membrane exerting a true barrier effect against rapidly proliferating epithelial cells [46,47]. The thickness values allow us to evaluate the barrier capacity since the membrane must maintain a minimum thickness during the healing period to be able to achieve the desired cell exclusion [12]. In this investigation, JM obtained the best results against the different degradation tests. It exceeded 21 days of immersion in PBS and 24 h of immersion in *C. histolyticum.* When the membrane was functionalized with zinc-ions or doxycycline (Zn-JM or Dox-JM), its resistance to degradation did not seem to improve. On the contrary, when Collprotect^®^ (CM) was functionalized (Zn-CM and Dox-CM), it obtained slightly better results in the biodegradation tests. One approach to extend the barrier function of membranes has consisted of functionalizing them or loading them with bioactive elements [33,34]. The affinity and strong adhesion of bacteria to collagen has been demonstrated in several investigations [48]. This means that upon exposure of the membrane to bacteria, contamination occurs, resulting in its rapid degradation [18], as presented with the *C. histolyticum* test. Therefore, functionalizing the membranes with an antibacterial agent can help overcome the problem of accelerated degradation due to bacterial contamination; this is an approach that has been chosen by several researchers. Chen et al. [49] coated a collagen membrane with silver nanoparticles, providing excellent antibacterial activity against *Staphylococcus aureus* and *Pseudomonas aeruginosa*. In this study, the membranes have been doped with zinc-ions and doxycycline. Wu et al. [50] found low rates of degradation when the membrane was funcionalized with zinc-ions. Bueno et al. [32] conducted a study in which they doped membranes, finding that doxycycline had a significant influence on the count of *S. oralis, V. parvula, A. naeslundii, F. nucleatum, A. actinomycetemcomitans* and *P. gingivalis* in the in vitro subgingival biofilm model. Toledano-Osorio et al. [51] carried out an in vitro investigation in which they demonstrated that doping collagen-based membranes for GBR with doxycycline significantly enhanced the proliferation and differentiation rates of cultured osteoblasts. In this paper, the addition of antimicrobial agents has not resulted in significant improvements in degradation rates. However, it could be concluded that functionalization with agents such as ionic zinc or doxycycline does not alter the membrane degradation pattern, adding the aforementioned benefits regarding its bioactivity.

In this study, situations that were more aggressive for the membranes than submerged healing were proposed through two degradation tests. Thanks to these tests, we can evaluate, in vitro, the possible attitude of the membranes in the worst scenario, which clinically would be in the presence of wound dehiscence. If resorbable membranes are exposed to the oral environment, the presence of saliva or collagenase derived from periodontal pathogens will rapidly degrade them [52]. The response, in terms of degradation, of the membranes was significantly greater when the specimens were subjected to the collagenase bacterial degradation test. When the samples were submerged in *C. histolyticum*, no membrane was able to exceed 48 h of immersion. JM resisted 24 h of immersion without completing 48 h, just like when it was functionalized with zinc-ions or doxycycline. The same occurred with CM which, both unfunctionalized and functionalized, did not exceed 24 h of immersion in the most aggressive medium. After analyzing the results obtained in this study, it is not possible to affirm that the addition of antimicrobial agents increases membranes’ resistance to bacterial degradation. A better response could be expected from the doped membranes if they were confronted with the microorganism instead of the toxin present in the medium. The hydrolytic degradation test was less aggressive with the membranes studied. Therefore, the resistance to degradation of the membranes in PBS was significantly greater while the degradation time of the membranes in *Clostridium* was significantly shorter. Taking this into account, the second null hypothesis must be rejected.

It could be stated that doping did not alter the membranes’characteristics, as the comparison between functionalized and non-functionalized membranes did not show significant differences in terms of degradation patterns. However, the results obtained in this research should be interpreted with caution due to its limitations. Some aspects, such as the correlation between the topography, nanostructure, mechanical properties, and influence of embedded cells on membrane degradation, have not been studied. Our in vitro findings aim to give a first estimate of the membranes’ behavior, but it must also be accepted that in vivo, the membrane degradation process can be influenced by different cell types, body fluids, and possible microbial contamination [42,43]. The structural changes that occurred on the degraded membranes were not analyzed in this study, which may be considered as a study limitation. However, it has been previously shown that the impact of collagen degradation on GBR membranes involves the alteration of the fibrous structure, mainly by the appearance of porous areas wider than 100 μm. These changes were not specific to the different membranes or even to the several performed degradation tests [12,15]. Therefore, it could be risky to directly transfer these data to clinical practice. For future research, an ambitious strategy could be to evaluate the tissue reaction and regenerative capability of these doped membranes in in vivo models, which may influence in the response of biodegradative membranes.

## 5. Conclusions

This study provides valuable in vitro results regarding the degradation of commercially available membranes. This contributes to advancing the frontiers of related knowledge and can inform surgeons’ decisions when selecting a specific membrane.

The studied membranes did not show the same degradation rate over time and did not behave in the same way in both degradation tests. Jason^®^, a native collagen membrane obtained from porcine pericardium, presented the highest resistance to the hydrolytic and bacterial collagenase degradation challenges. The functionalization of the membranes did not impair resistance to degradation. The bacterial collagenase solution acted more aggressively against the membranes.

These findings support the potential use of functionalized membranes as carriers for antibacterial medicaments, such as zinc or doxycycline, known for their benefits in the bone healing process. These outcomes lay the groundwork for future clinical studies, encouraging researchers to clinically test doped membranes in GBR or GTR procedures where the effect of functionalization could influence the regenerative events.

## Figures and Tables

**Table 1:** Main properties of the collagen membranes tested in the study.

Attribute	Jason^®^ Membrane	Collprotect^®^ Membrane
Origin	Porcine pericardium	Porcine dermis
Composition	Native collagen type III	Native collagen type I and III
Crosslinking	No cross-linked	Naturally cross-linked
Structure	Natural multilayered collagen structure	Natural 3D collagen structure, rough and porous
Thickness	0.05–0.35 mm	0.4 mm
Degradation time	Slow degradation rate 12–24 weeks	Intermediate degradation rate 4–8 weeks

**Table 2:** Thickness evaluation of the studied membranes subjected to the degradation tests over the different periods of time. (**a**) The measurements (means and standard deviations (mm)) of the membranes (JM, Zn-JM, Dox-JM, CM, Zn-CM and Dox-CM) at the different times periods (baseline to 21days) in the two degradation tests (PBS and *C. histolyticum* collagenase). (**b**) *p* values from Bonferroni’s correction for pairwise comparisons between the membranes’ thicknesses at different time periods and the baseline thickness (Th at t0: Th0). Statistical significance was set at *p* ≤ 0.001.

(**a**)												
	**JM**		**Zn-JM**		**Dox-JM**		**CM**		**Zn-CM**		**Dox-CM**	
	PBS	*C.H*	PBS	*C.H*	PBS	*C.H*	PBS	*C.H*	PBS	*C.H*	PBS	*C.H*
t0	0.021 (0.027)	0.041 (0.004)	0.021 (0.018)	0.018 (0.025)	0.024 (0.019)	0.012 (0.016)	0.122 (0.048)	0.078 (0.089)	0.056 (0.056)	0.060 (0.076)	0.041 (0.048)	0.063 (0.048)
12 h	0.056 (0.021)	0.034 (0.004)	0.044 (0.010)	0.029 (0.005)	0.0038 (0.006)	0.024 (0.005)	0.140 (0.053)	0.069 (0.058)	0.111 (0.085)	0.046 (0.037)	0.132 (0.027)	0.060 (0.048)
24 h	0.065 (0.011)	0.079 (0.095)	0.043 (0.010)	0.054 (0.131)	0.043 (0.036)	0 (0)	0.213 (0.100)	0 (0)	0.083 (0.090)	0 (0)	0.114 (0.069)	0 (0)
48 h	0.056 (0.009)	0 (0)	0.039 (0.005)	0 (0)	0.053 (0.029)	0 (0)	0.132 (0.014)	0 (0)	0.102 (0.078)	0 (0)	0.190 (0.055)	0 (0)
7 d	0.052 (0.005)	0 (0)	0.048 (0.014)	0 (0)	0.042 (0.011)	0 (0)	0 (0)	0 (0)	0 (0)	0 (0)	0 (0)	0 (0)
21 d	0.016 (0.023)	0 (0)	0.009 (0.013)	0 (0)	0.011 (0.017)	0 (0)	0 (0)	0 (0)	0 (0)	0 (0)	0 (0)	0 (0)
(**b**)												
0–12 h	0.008	0.003	0.003	0.207	<0.001	0.889	0.320	0.023	0.172	0.623	<0.001	<0.001
0–24 h	<0.001	0.251	0.005	0.434	0.020	0.001	0.026	0.018	0.505	0.032	0.020	0.001
0–48 h	0.002	<0.001	0.010	0.045	<0.001	0.001	0.566	0.018	0.227	0.032	<0.001	0.001
0–7 d	0.004	<0.001	0.002	0.045	0.021	0.001	<0.001	0.018	0.040	0.032	0.021	0.001
0–21 d	0.668	<0.001	0.124	0.045	0.021	0.001	<0.001	0.018	0.040	0.032	0.021	0.001

JM: Unfunctionalized Jason^®^ membrane; Zn-JM: Jason^®^ functionalized with zinc-ions; Dox-JM: Jason^®^ functionalized with doxycycline; CM: Unfunctionalized Collprotect^®^ Membrane; Zn-CM: Collprotect^®^ functionalized with zinc-ions; Dox-CM: Collprotect^®^ functionalized with doxycycline; PBS: Phosphate Buffer Saline; CH: *C. histolyticum.*

**Table 3:** Weight measurements of the studied membranes subjected to degradation tests over the different periods of storage time. (**a**) W measurements (means and standard deviations (g)) of the membranes (JM, Zn-JM, Dox-JM, CM, Zn-CM and Dox-CM) at the different time periods (baseline to 21 days) in the two degradation tests (PBS and *C. histolyticum* collagenase). (**b**) *p* values from Bonferroni’s correction for pairwise comparisons between membranes’ weights at the different time periods and at the baseline weight (W at t0: W0). Statistical significance was set at *p* ≤ 0.001.

(**a**)												
	JM		Zn-JM		Dox-JM		CM		Zn-CM		Dox-CM	
	PBS	*C.H*	PBS	*C.H*	PBS	*C.H*	PBS	*C.H*	PBS	*C.H*	PBS	*C.H*
t0	0.006 (0)	0.005 (0.001)	0.003 (0)	0.003 (0)	0.003 (0)	0.003 (0)	0.010 (0.001)	0.009 (0.001)	0.006 (0.005)	0.038 (0.044)	0.009 (0.001)	0.009 (0.001)
12 h	0.006 (0)	0.004 (0)	0.003 (0)	0.002 (0)	0.003 (0)	0.002 (0)	0.009 (0.001)	0.004 (0.003)	0.006 (0.004)	0.002 (0.001)	0.008 (0.001)	0.001 (0.002)
24 h	0.005 (0.001)	0.002 (0.001)	0.003 (0)	0.001 (0.001)	0.003 (0)	0 (0)	0.008 (0.001)	0 (0)	0.005 (0.004)	0 (0)	0.007 (0.001)	0 (0)
48 h	0.005 (0)	0 (0)	0.003 (0)	0 (0)	0.003 (0)	0 (0)	0.007 (0)	0 (0)	0.003 (0.003)	0 (0)	0.007 (0.001)	0 (0)
7 d	0.005 (0)	0 (0)	0.004 (0.001)	0 (0)	0.003 (0)	0 (0)	0 (0)	0 (0)	0 (0)	0 (0)	0 (0)	0 (0)
21 d	0.002 (0.003)	0 (0)	0.002 (0.003)	0 (0)	0.001 (0.002)	0 (0)	0 (0)	0 (0)	0 (0)	0 (0)	0 (0)	0 (0)
(**b**)												
0–12 h	0.304	0.002	0.084	<0.001	0.035	<0.001	0.070	<0.001	0.116	0.025	0.035	<0.001
0–24 h	0.064	<0.001	0.885	<0.001	<0.001	<0.001	0.003	<0.001	0.591	0.019	<0.001	<0.001
0–48 h	<0.001	<0.001	1.000	<0.001	<0.001	<0.001	<0.001	<0.001	0.118	0.019	<0.001	<0.001
0–7 d	0.001	<0.001	0.106	<0.001	<0.001	<0.001	<0.001	<0.001	0.001	0.019	<0.001	<0.001
0–21 d	<0.001	<0.001	0.107	<0.001	<0.001	<0.001	<0.001	<0.001	0.001	0.019	<0.001	<0.001

JM: Unfunctionalized Jason^®^ membrane; Zn-JM: Jason^®^ functionalized with zinc-ions; Dox-JM: Jason^®^ functionalized with doxycycline; CM: Unfunctionalized Collprotect^®^ Membrane; Zn-CM: Collprotect^®^ functionalized with zinc-ions; Dox-CM: Collprotect^®^ functionalized with doxycycline; PBS: Phosphate Buffer Saline; CH: *C. histolyticum.*

## Data Availability

The data presented in this study are available upon request from the corresponding author.

## Abbreviations

CH	*C. histolyticum*
CM	Unfunctionalized Collprotect^®^ Membrane
Dox-CM	Collprotect^®^ functionalized with doxycycline
Dox-JM	Jason^®^ functionalized with doxycycline
GBR	Guided Bone Regeneration
GTR	Guided Tissue Regeneration
JM	Unfunctionalized Jason^®^ membrane
MMP	Matrix Metalloproteinase
MNGCs	Multinucleated Giant Cells
PBS	Phosphate Buffer Saline
Th	Thickness
W	Weight
Zn-CM	Collprotect^®^ functionalized with zinc-ions
Zn-JM	Jason^®^ functionalized with zinc-ions
